# Comparing ovarian reserve parameters after laparoscopic endometrioma resection in the follicular vs. luteal phase: a prospective cohort study

**DOI:** 10.3389/fmed.2024.1469858

**Published:** 2024-10-15

**Authors:** Ozguc Takmaz, Mehmet Resit Asoglu, Mehmet Ceyhan, Gozde Unsal, Mete Gungor

**Affiliations:** ^1^Department of Obstetrics and Gynecology, Maslak Hospital, Acibadem Mehmet Ali Aydinlar University, School of Medicine, İstanbul, Türkiye; ^2^Department of Obstetrics and Gynecology, Bahceci Fertility Clinic, Üsküdar University, İstanbul, Türkiye

**Keywords:** endometrioma removal, ovarian damage, anti-Mullerian hormone, endometriosis, ovarian reserve

## Abstract

**Objective:**

To evaluate whether performing laparoscopic endometrioma surgery in the follicular or luteal phase affects changes in ovarian reserve.

**Methods:**

This prospective cohort study was conducted in a university-affiliated hospital. A total of 55 women who underwent unilateral laparoscopic endometrioma removal were included in the study. Of these, 28 were in the follicular phase of the menstrual cycle (follicular group), and 27 were in the luteal phase (luteal group). The primary outcomes were the rates of decreased anti-Mullerian hormone (AMH) levels and antral follicle counts (AFC) 6 months after the surgery, which were compared between the groups.

**Results:**

The groups were similar in patient characteristics and endometrioma sizes. AMH levels and AFCs were significantly lower in the post-operative 6th month compared with their pre-operative values (*p* < 0.05 for both groups). The rate of decrease in AMH levels 6 months after the surgery was not significantly different between the follicular and luteal groups (24.5 and 19.5%, respectively, *p* > 0.05). Similarly, the rate of decrease in AFCs 6 months after the surgery did not differ between the groups (13.4 and 14.3%, respectively, *p* > 0.05).

**Conclusion:**

Performing laparoscopic endometrioma surgery, whether in the follicular or luteal phase, does not seem to affect the changes in the ovarian reserve.

**Clinical trial registration:**

https://clinicaltrials.gov/study/NCT03484546, identifier NCT034845.

## Introduction

Many studies have shown that endometrioma surgery decreases ovarian reserve (1–3). However, the definitive treatment of endometrioma-related dysmenorrhea, dyspareunia, and suspicious adnexal masses is still the surgical removal of the endometriomas (4). Although there is controversy regarding the indications for endometrioma removal, it remains a frequently performed surgical procedure to relieve symptoms.

Since endometrioma negatively affects ovarian reserve and endometriosis is a known cause of infertility, preserving ovarian reserve during endometrioma surgery may be more critical than other ovarian cyst surgeries. Furthermore, endometrioma patients have a higher risk of recurrence and the necessity for repetitive surgeries compared to other ovarian cysts.

Studies indicate that surgical technique and the surgeon’s experience are important factors in reducing the negative effect of endometrioma surgery on ovarian reserve (5). Alternative surgical techniques have been described in these studies. The main goal of these techniques is to minimize damage to healthy follicles during hemostatic procedures. Some of these techniques include suturing instead of using electrocoagulation, using bipolar electrosurgical instruments instead of monopolar, vasopressin injection, and the use of hemostatic sealants (6–8). Although these techniques are partially effective, they still do not seem to be sufficient to reduce the changes in ovarian reserve.

Few studies have investigated other factors affecting the changes in ovarian reserve. One of these factors is the menstrual cycle phase during endometrioma surgery. Studies have observed differences in the histological and vascular findings of ovaries based on the menstrual cycle phases (9, 10). A study recently published by Wu et al. (11) found that performing endometrioma surgery during the late luteal phase reduces changes in ovarian reserve.

In our study, we investigated whether the extent of damage to the ovarian reserve is affected by the menstrual cycle phases in laparoscopic endometrioma removal.

## Material methods

This prospective study was conducted in the Department of Gynecology of Acibadem Mehmet Ali Aydinlar University Maslak Hospital from 28 March 2018 through 1 October 2021 and approved by the Institutional Ethics Committee (ATADEK, ID no: 2018–4/18). Written informed consent was obtained from all participants. The study was registered at Clinicaltrials.gov (ID No: NCT03484546). https://clinicaltrials.gov/study/NCT03484546

All procedures followed the ethical standards of the 1964 Declaration of Helsinki and its later amendments or comparable ethical standards.

### Study design

Patients diagnosed with unilateral endometrioma by ultrasound, aged between 18 and 40 years, with regular menstrual periods, and with indications for endometrioma removal were enrolled in the study. Indications for the surgery were dysmenorrhea, dyspareunia, increase in the size of cyst diameter and the suspicion adnexal mass other than endometrioma. The exclusion criteria were as follows: the presence of non-endometrioma cyst (according to pathology results), bilateral or multiple cyst removal, an additional surgical procedure in the same session, a history of previous ovarian surgery, pre- or post-operative hormonal medication use (oral contraceptives, gonadotropin analogs/antagonists, progestins), chronic anticoagulant use (possible excessive hemostatic intervention), deep infiltrating endometriosis and the presence of dense adhesions between endometrioma and intraabdominal structures (Severe—Stage IV Revised-ASRM endometriosis classification (12)), pregnancy within 6 months after surgery, having irregular menstrual periods, premature ovarian failure and post-menopausal status (Table 1).

**Table 1:** Patient exclusion criteria.

Exclusion criteria
Non-endometriomal cyst (according to final pathology report)
Bilateral and/or multiple cyst removal
Additional surgical procedure in the same session
Previous ovarian surgery
Oral contraceptive, gonadotrophic releasing hormone agonists or progestin use in pre and post-operative period
Chronic anti-coagulant use before operation
Deep infiltrating endometriosis and severe adhesions between endometrioma and intraabdominal structures (Severe Stage IV r-ASRM endometriosis classification)
Pregnancy within 6 months after the surgery
Irregular menstrual periods whose menstrual phases cannot be determined
Post-menopausal status, premature ovarian failure

**Figure 1:**
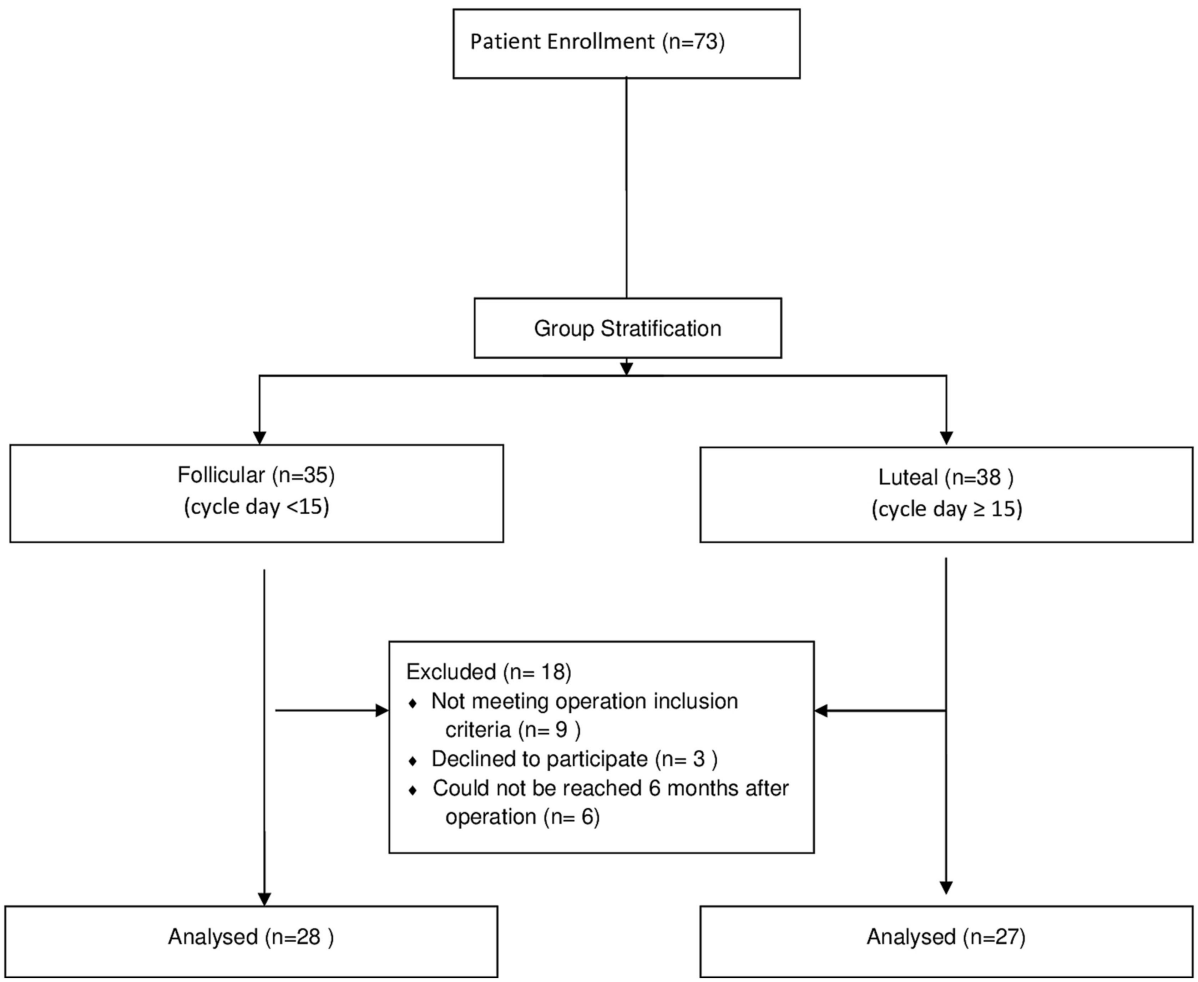
Study flow diagram.

The menstrual day of the patients on the operation day was calculated by adjusting to a 28-day cycle using a formula described by Ramakrishan et al. (13) and Song et al. (14). The adjusted day of the menstrual cycle = (14 x day of the cycle at the time of surgery) / (cycle length of the patient - 14). Patients whose adjusted cycle day <15 were grouped as follicular, while those with an adjusted cycle day ≥15 were grouped as luteal (Figure 1).

When our study was designed, there were no comparable studies in the existing literature. In determining the sample size for power of our study, we examined other studies exploring the impact of different surgical techniques in endometrioma cystectomy on ovarian reserve. In the study conducted by Song et al. (7), the preoperative and postoperative percentage changes in AMH levels were evaluated based on two surgical techniques (Bipolar Coagulation group: 42.2%; Suture group: 24.6%). The calculation yielded an effect size of *d* = 0.799, and it was determined that a minimum of 26 individuals per group is necessary to achieve 80% power at the 0.05 significance level. The G*Power program (Heinrich Heine Universität Dusseldorf, 2020) was used to calculate the sample size.

Diameter measurements of endometriomas and antral follicle counts of the same ovary were performed using transvaginal ultrasound and recorded in the outpatient service. Ultrasonography were performed vaginally by the same physician (OT) with GE Voluson S8 (GE Health Care, Chicago, United States) via wideband microconvex endocavitary 180^0^ 2.9–9.7 MHz probe. The operation day was scheduled based on operating room availability and patient preferences. The assignment of participants to study groups based on surgery during the follicular vs. luteal phase was nonrandomized, thus our study is not a randomized controlled study. On the surgery day, blood samples were collected for pre-operative AMH levels in the inpatient service. The serum was separated by centrifugation and stored at −70°C.

All operations were performed using the same technique by two experienced surgeons (OT, MG) in minimally invasive gynecologic surgery. Operations were performed via four abdominal ports (10 mm umbilical, 5 mm right, left, and suprapubic ports). Ovarian surfaces were incised, and endometrioma cysts were removed using the stripping technique. Hemostasis was achieved with bipolar forceps coagulation adjusted to 30 W power. No suturing or hemostatic agents were used. Cysts were removed from the abdominal cavity in a contained bag system. Operating time was defined as the time from the first skin incision to the end of skin closure. Estimated blood loss (EBL) was calculated as the difference in fluid volume between irrigation and suction.

Patients whose diagnosis of endometrioma was confirmed in the pathology reports were asked to be followed up 6 months after the surgery. Antral follicle counts were performed on the operated ovary with ultrasonography. Subsequently, blood samples were collected for post-operative AMH levels. The serum was separated and stored at −70°C.

After patient enrollment was completed, serum samples were thawed. AMH levels were measured with an enzyme immunosorbent assay kit (Ansh Labs, Webster, TX, United States) according to the manufacturer’s instructions. Patient characteristics (age, BMI, gravida, endometrioma diameter, antral follicle count) and surgery data (operation time, EBL) were obtained from the study data records.

### Outcome measures

The primary outcome was the decrease rate in AMH levels and AFCs 6 months after the surgery. The decrease rate of serum AMH levels was defined as: Rate of decline (%) = 100 × (Preoperative AMH level − Postoperative AMH level) / Preoperative AMH level. The decrease rate of AFC was calculated as: Rate of decline (%) = 100 × (Preoperative AFC − Postoperative AFC / Preoperative AFC). The secondary outcome was the estimated blood loss in the surgery.

### Statistical analysis

All analyses were conducted in NCSS (Number Cruncher Statistical System) 2020 Statistical Software (NCSS LLC, Kaysville, Utah, United States). Quantitative variables were shown with mean, standard deviation, median, and quartile values, and qualitative variables were shown with descriptive statistical methods such as frequency and percentage. The assumption of normality was made with Shapiro–Wilk and Kolmogorov–Smirnov tests. Normal distributed data were evaluated with the Student *T*-Test, while non-normally distributed data was assessed with the Mann–Whitney *U* and Wilcoxon Signed Rank test. Decrease rate of AMH and AFC were compared with Mann–Whitney *U* test. Spearman’s rank correlation coefficient was used to assess the correlation between menstrual cycle day and the decreased rate of AMH levels. *p* value of <0.05 was considered as the threshold for statistical significance.

## Results

Of the 73 enrolled patients, 18 were excluded because they did not meet the inclusion criteria [kissing ovaries (5), non-endometrial cysts (3), bilateral cystectomy (1), declined to participate (3), could not be reached after the operation (6)]. Fifty-five patients met the inclusion criteria; 28 were in the follicular group, and 27 were in the luteal group (Figure 1).

Age, body mass index (BMI), gravida, endometrioma diameter, operative time, and estimated blood loss did not differ statistically between the groups (Table 2). The median pre-operative AMH levels were comparable between the groups (2.4 ng/mL (1.3–4.6) and 2.8 ng/mL (1.9–4.0) respectively, *p* = 0.67). Post-operative AMH levels at 6 months were significantly lower than pre-operative AMH levels for both the follicular and luteal groups (*p* = 0.001 for both groups). Additionally, post-operative AFCs at 6 months were significantly lower than pre-operative AFC for both groups (*p* = 0.03 and *p* = 0.002, respectively).

**Table 2:** Patient characteristics.

	Follicular (*n* = 28)	Luteal (*n* = 27)	*p*
Age	29.96 ± 7.01	31.07 ± 6.57	^**a**^ **0.54**
BMI ^c^(kg/m^2^)	23.3 ± 3.7	22.01 ± 3.45	^**a**^ **0.17**
Gravida	0 (0–1)	0 (0–0)	^**b**^ **0.93**
Endometrioma Diameter (cm)	6 (4.6–7.8)	6 (5–7)	^**b**^ **0.47**
Operation time (min)	62.04 ± 12.93	57.96 ± 15.77	^**a**^ **0.29**
EBL^d^ (ml)	47.5 (40–53.8)	50 (45–55)	^**b**^ **0.32**

Data are given as mean ± standard deviation, median (Q1–Q3), Q1–Q3: %25–%75 percentiles.

^aStudent-t Test; bMann Whitney-U Test; cBody Mass Index; dEstimated blood loss.^

The rates of decrease in AMH levels for the follicular and luteal groups (24.5 and 19.5%, respectively) were comparable (*p* = 0.52).

The changes in AFC 6 months after the surgery were similar between the groups, as well [13.4% (−9.4–25.0) and 14.3 (0–28.6), respectively, *p* = 0.54] (Table 3).

**Table 3:** Comparison of pre-post operative AMH, AFC, and decrease rates.

	Pre-operative AMH (ng/ml)	Post-operative AMH (ng/ml)	P(for pre-post AMH)	Pre-operative AFC	Post-operative AFC	P(for pre-post AFC)	Decrease rate of AMH (%)	Decrease rate of AFC (%)
**Follicular** **(*n* = 28)**	2.4 (1.3–4.6)	2.01 (0.9–3.6)	^**a**^ **0.001**	7 (6-8)	6.5 (5–7)	^**a**^ **0.03**	24.5 (12.8-48.3)	13.4 (−9.4–25.0)
**Luteal** **(*n* = 27)**	2.8 (1.9–4.0)	2.07 (1.2–3.9)	^**a**^ **0.001**	7 (6–8)	6 (5–7)	^**a**^ **0.002**	19.5 (7.9–35.0)	14.3 (0–28.6)
***p***	^**b**^ **0.67**	^**b**^ **0.82**		^**b**^ **0.67**	^**b**^ **0.83**		^**b**^ **0.52**	^**b**^ **0.54**

Data are given as median (Q1–Q3), Q1–Q3: %25–%75 percentiles.

^aWilcoxon Signed Rank test; bMann Whitney-U Test.^

In addition, AMH difference rates did not correlate with menstrual cycle day for both the follicular and luteal groups (*p* = 0.68, *p* = 0.43, *r* = −0.08, *r* = −0.15, respectively).

## Discussion

In our study, we prospectively evaluated the effect of being in the follicular or luteal phase on the day of surgery on ovarian reserve in patients who underwent laparoscopic endometrioma surgery. This study concluded that the menstrual phase itself did not significantly affect the extent of changes in ovarian reserve, measured through AMH levels and AFCs. The estimated blood loss was also not affected by the menstrual phase during surgery. In addition, there was no correlation between the cycle day and the degree of changes in ovarian reserve.

Damage to healthy follicles in endometrioma surgery occurs in two stages: the excision of the healthy cortical tissue while removing the cyst and the injury to healthy follicles during hemostasis. Alternative surgical techniques have been recommended to minimize the damage to healthy follicles. Suturing instead of electro-coagulation and using bipolar energy instead of monopolar for hemostasis are some of recommended techniques (6, 15). In addition, using hemostatic sealants can also be beneficial (16).

A limited number of studies have investigated variables other than surgical technique to reduce ovarian damage in women undergoing laparoscopic ovarian surgery. One possible variable that may alter the extent of ovarian damage is the menstrual cycle phase on the day of surgery.

Some studies claimed that operating on different days of the menstrual cycle can change the amount of blood loss during surgery. Paraskevaidis et al. found increased blood loss when the loop electrosurgical excision procedure (LEEP) was performed in the luteal phase of the menstrual cycle (17). Similarly, Sariguney et al. and Findikcioglu et al. observed a significant increase in blood loss when mammoplasty and rhinoplasty were performed in the luteal phase (18, 19). In contrast, other studies found no significant relationship between the menstrual cycle phase on the day of surgery and blood loss (20–23).

In our opinion, it is less likely that non-gynecologic organs are affected by the phase of the menstrual cycle. However, gynecological organs, whose functions and structures differ during the menstrual cycle, may be affected by the menstrual cycle changes. It has been well documented that the blood flow of the uterus and ovaries varies with cyclic hormonal changes (24). Sladkevicius et al. showed that the pulsatility index and time-averaged maximum velocity were lower during the menstrual period in the dominant ovary (25). In a study using Doppler ultrasonography throughout the cycle conducted by Tan et al., FSH levels and the blood supply of the ovary with the dominant follicle increased, while there was no change in Doppler findings in the non-dominant ovary (24). These studies suggest that ovarian blood flow varies within the different cycle phases.

In the study by Song et al., medical records of 155 patients were reviewed. They retrospectively concluded that the menstrual cycle phase during surgery did not affect ovarian damage and was not an essential factor in determining the optimal time for ovarian cystectomy (14). This study had some limitations. The patients had different gynecologic conditions (dermoid, endometrioma, other). The cases that underwent bilateral cystectomy were also enrolled in this study, which might have caused more injury to the ovarian reserve than those undergoing unilateral cystectomy. Another limitation was that post-operative AMH levels were measured 3 months after the operations. In studies that measured AMH levels after ovarian surgery, it was found that AMH levels recovered in the 6th month after surgery. Thus, in our study, post-operative AMH levels were measured 6 months after the operations (3).

Wu et al. conducted a randomized controlled study on the subject. They found that performing laparoscopic endometrioma removal in the late luteal phase significantly reduces ovarian damage. In their study, patients were given oral contraceptives (OC) to determine the late luteal and early follicular phase groups. Although the prospective randomized design strengthens this study, administering OC could inhibit ovulation, potentially preventing regular physiological changes in the ovaries related to blood flow and histology. In our study, patients were grouped based on their natural cycles.

Our study has strengths and limitations. On the positive side, our study was prospective and high-powered. All surgeries were performed by the same surgeons using a standard technique, and patients’ cycle phases were determined by optimizing their natural menstrual cycles. However, our study also has limitations. We could not confirm that the operated ovary led to the dominant follicle when the surgery was performed, as no ultrasound follow-up or ovulation tests were done during that menstrual cycle. Additionally, we did not consider in which phase of the cycle the patients’ preoperative and postoperative AFCs were performed. The main reason for this was that AMH levels are not affected by cycle variations. While suturing is recommended over electro-coagulation, it was not performed in our study’s surgical technique. Nevertheless, we believe our study results were not affected since the same technique was consistently used for all patients. In addition, changes in AMH levels and AFC are used for assessment of ovarian reserve changes, they are not always concordant with clinical ovarian reserve. Furthermore, although our study had high power, the sample size was relatively small.

In conclusion, the menstrual phase on the day of surgery does not significantly affect ovarian reserve damage during laparoscopic endometrioma removal. It suggests that surgeons may not need to consider menstrual cycle phases when scheduling these surgeries, allowing for more flexibility and convenience for both patients and physicians.

## Data Availability

The study’s raw data is available at: https://osf.io/mry73/.
